# An Explainable AI Tool (FibroX) for Detecting Advanced Liver Fibrosis in Adults With Type 2 Diabetes: Protocol for a Pilot Crossover Trial

**DOI:** 10.2196/90456

**Published:** 2026-05-21

**Authors:** Basile Njei, Ulrick Sidney Kanmounye

**Affiliations:** 1 Section of Digestive Diseases Department of Medicine Yale University New Haven, CT United States; 2 Engelhardt School of Global Health and Bioethics Euclid University Bangui Central African Republic; 3 VA Connecticut Healthcare West Haven, CT United States; 4 Ohio University Heritage College of Osteopathic Medicine Athens, OH United States; 5 Yale Liver Center Yale New Haven Health New Haven, CT United States; 6 International Medicine Program Yale University New Haven, CT United States; 7 Association of Future African Neurosurgeons Yaounde Cameroon

**Keywords:** artificial intelligence, AI, liver fibrosis, metabolic dysfunction–associated steatotic liver disease, MASLD, pilot, randomized controlled trial, type 2 diabetes

## Abstract

**Background:**

Metabolic dysfunction–associated steatotic liver disease is highly prevalent in adults with type 2 diabetes, and advanced fibrosis is its strongest prognostic marker. However, existing noninvasive tools may underperform in diabetes care and are inconsistently used in practice.

**Objective:**

This study aims to evaluate the feasibility, usability, and preliminary diagnostic effectiveness of FibroX, an explainable artificial intelligence tool for identifying metabolic dysfunction–associated steatotic liver disease–associated advanced fibrosis in adults with type 2 diabetes.

**Methods:**

This 12-month health care provider–level randomized crossover pilot trial will enroll at least 36 primary care clinicians managing adults with type 2 diabetes. Participants will complete 2 simulated care periods: one with FibroX-enabled care and one with usual care, separated by a 1-week washout. FibroX generates individualized advanced fibrosis risk estimates from routine clinical data, provides guideline-aligned triage categories, and displays case-level explanatory outputs using Shapley Additive Explanations. FibroX is an investigational tool and is not currently used in routine clinical practice. Primary outcomes are feasibility and usability, while secondary and exploratory outcomes include workflow efficiency, preliminary diagnostic performance, and implementation measures.

**Results:**

Institutional review board approval has been obtained. The protocol was registered on ClinicalTrials.gov (NCT07305324) on December 1, 2025. At the time of submission, recruitment had not yet begun. The study is currently unfunded. Preparatory activities, including platform development and case validation, are ongoing. Recruitment is expected to begin in June 2026, with primary completion anticipated in May 2027 and results expected to be available in late 2027.

**Conclusions:**

This pilot study will provide preliminary evidence on the feasibility, usability, and diagnostic performance of an explainable artificial intelligence tool for fibrosis risk stratification in diabetes care and will inform the design of a future larger trial.

**Trial Registration:**

ClinicalTrials.gov NCT07305324; https://clinicaltrials.gov/study/NCT07305324

**International Registered Report Identifier (IRRID):**

PRR1-10.2196/90456

## Introduction

### Background

Metabolic dysfunction–associated steatotic liver disease (MASLD) is highly prevalent and clinically consequential among people living with type 2 diabetes (T2D). Population studies indicate that steatosis and progressive fibrosis affect a large proportion of adults with T2D, with MASLD prevalence approaching 70% in some cohorts, and advanced fibrosis (stage ≥F3) conferring markedly elevated risks of liver-related events and cardiovascular morbidity and mortality [1-4]. Advanced fibrosis is the strongest prognostic marker in MASLD and is associated with 2 to 3-fold higher liver-related and cardiovascular mortality in diabetes populations [4,5]. Recent diabetes-focused guidance has therefore emphasized that adults with prediabetes or type 2 diabetes are a priority population for structured noninvasive fibrosis risk stratification rather than opportunistic case finding alone [2,3]. However, real-world implementation in primary care and diabetes clinics remains inconsistent, leading to missed opportunities for early intervention [1-4].

Current approaches to fibrosis assessment rely on a mix of laboratory-based composite scores (eg, the Fibrosis-4 Index [FIB‑4] and nonalcoholic fatty liver disease fibrosis score) and imaging modalities (eg, vibration-controlled transient elastography [VCTE] and magnetic resonance elastography) [6-8]. While elastography provides strong diagnostic performance, it requires dedicated equipment and trained personnel, limiting availability; meanwhile, fixed-formula laboratory scores can underperform in T2D populations where metabolic and inflammatory profiles differ, and they do not capture complex, nonlinear relationships among routinely collected clinical variables [9-12]. Liver biopsy, although a reference standard, is invasive, subject to sampling variability, and not suitable for screening at scale [6]. Consequently, many individuals with T2D and advanced fibrosis remain undetected until late disease stages, when therapeutic options are limited and outcomes are poorer [7,13]. In real-world practice, these pathways often depend on simplified cutoffs followed by second-line elastography, which can leave many at-risk patients in indeterminate categories and create access and workflow barriers in primary care [4,14,15].

Explainable artificial intelligence (AI) offers a path to more accurate, transparent, and workflow-compatible decision support in diabetes care. Prior work has shown that machine learning models leveraging routinely available data can improve discrimination for high-risk MASLD and fibrosis outcomes, while post hoc explanation methods enhance interpretability and clinician trust [16-19]. Recent studies in liver disease and other clinical prediction settings have increasingly used explainable machine learning approaches to move beyond black-box risk estimates and provide patient-level feature attribution that may support clinician interpretation and adoption [20-22].

Building on this foundation, FibroX was developed to estimate individualized risk of advanced fibrosis (≥F3) using common clinical inputs (age, aspartate aminotransferase [AST], alanine aminotransferase [ALT], platelets, hemoglobin A_1c_ [HbA_1c_], BMI, creatinine or estimated glomerular filtration rate), with dual-threshold triage bands aligned to guidelines (rule out, indeterminate to elastography, and rule in to hepatology referral) and Shapley Additive Explanations (SHAP) to surface the features most responsible for each prediction [17,18]. In prior work, FibroX was derived using an explainable Extreme Gradient Boosting framework applied to population-based National Health and Nutrition Examination Survey data and subsequently evaluated in external biopsy-confirmed MASLD cohorts. Across these earlier evaluations, FibroX showed better discrimination for advanced fibrosis than conventional noninvasive scores such as FIB-4 and was also associated with prognostic stratification for longer-term outcomes. However, FibroX remains investigational, and its feasibility, usability, and influence on clinician decision-making have not yet been prospectively assessed in a clinical workflow context [15-18].

### Study Objectives and Rationale

**Figure 1:**
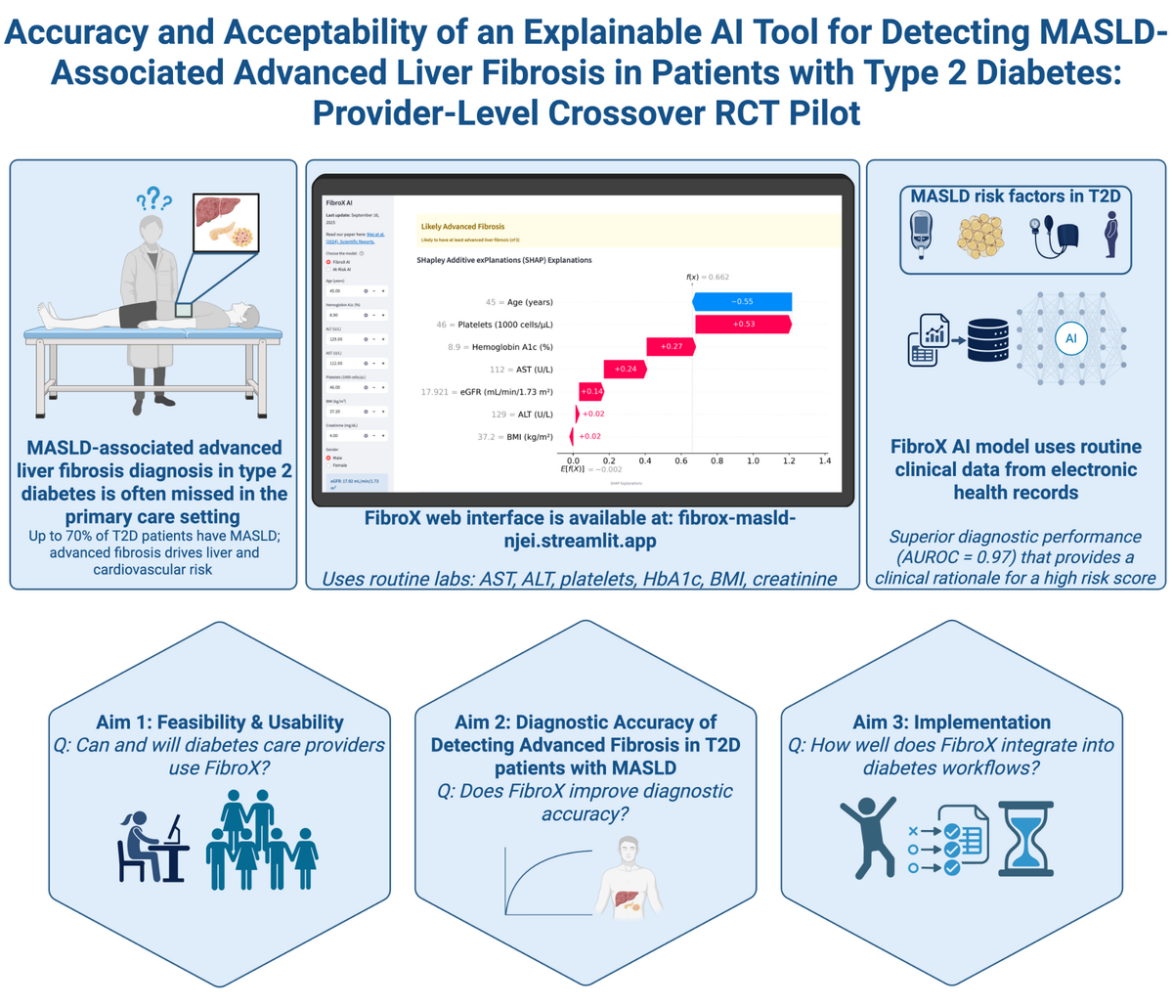
Schematic of FibroX inputs, explainable outputs, and clinician actions in the simulated diabetes care workflow. ALT: alanine aminotransferase; AST: aspartate aminotransferase; AUROC: area under the receiver operating characteristic curve; eGFR: estimated glomerular filtration rate; HbA_1c_: hemoglobin A1c; MASLD: metabolic dysfunction–associated steatotic liver disease; SHAP: Shapley Additive Explanations; T2D: type 2 diabetes.

This pilot aims to evaluate the feasibility, usability, and preliminary effectiveness of FibroX as an explainable AI decision support tool for detecting MASLD-associated advanced fibrosis in adults with T2D during simulated clinical encounters. Specifically, we aim to (1) assess the primary pilot end points of feasibility and usability in diabetes-focused primary care workflows using validated instruments, (2) estimate secondary outcomes related to workflow efficiency and preliminary diagnostic performance for advanced fibrosis, and (3) explore adoption and implementation metrics using the Reach, Effectiveness, Adoption, Implementation, and Maintenance framework, including guideline-concordant triage and net reclassification improvement (Figure 1).

If FibroX proves feasible and acceptable and improves diagnostic discrimination, it could help operationalize guideline-concordant triage, reduce missed diagnoses, and inform equitable MASLD screening strategies in diabetes care [1-4,10,12,13,16].

## Methods

### Trial Design and Oversight

We will conduct a 12-month, health care provider–level, randomized, 2‑period crossover simulation trial to evaluate the feasibility, usability, and preliminary effectiveness of FibroX, an explainable AI decision support tool for detecting MASLD‑associated advanced liver fibrosis (≥F3) in adults with T2D. Because FibroX is not currently used in routine clinical care, this study evaluates the tool in a simulated clinical environment. Each clinician serves as their own control (FibroX-enabled care vs usual care), separated by a 1-week washout. Distinct, nonoverlapping simulation case sets will be used in each study period, such that clinicians will not evaluate the same cases across periods, reducing case-recall bias and minimizing carryover related to prior exposure. The study involves no patient enrollment and uses deidentified or synthetic cases. Oversight is by the principal investigator and study steering team; a formal Data Monitoring Committee is not required given the minimal risk.

### Study Setting

Health care provider participants will be recruited from Yale-affiliated diabetes and primary care clinics and participating federally qualified health centers. Simulations are delivered via a secure, web-based platform designed to replicate an electronic health record (EHR)–like interface. Each simulated case presents standardized clinical information, including patient demographics, laboratory values, and relevant clinical notes. Participants review cases sequentially and make diagnostic and management decisions within this interface. In the FibroX-enabled condition, additional outputs—including predicted risk of advanced fibrosis, guideline-aligned triage categories, and SHAP-based feature explanations—are displayed alongside standard clinical data.

### Participants

This pilot will enroll licensed primary care clinicians, including physicians, nurse practitioners, and physician assistants. Inclusion criteria require health care providers to have ≥0.5 full-time equivalent dedicated to adult diabetes care at participating sites, the ability to complete 2 simulation periods, and provision of informed consent. Exclusion criteria include prior participation in any FibroX usability or validation studies, exclusive practice in pediatrics or nondiabetes specialty areas, or inability to complete all study procedures. Participating clinicians are expected to have varying levels of clinical experience, reflecting real-world primary care practice, but all are required to have active involvement in the management of adult patients with T2D to ensure familiarity with relevant clinical workflows and decision-making. Participants will be recruited through email invitations and departmental announcements across Yale-affiliated primary care and diabetes clinics, as well as participating federally qualified health centers. Recruitment will target clinicians with active involvement in the management of adults with T2D, with participation on a voluntary basis.

The simulation case library will comprise adult patients (aged ≥18 years) with T2D and at least one additional MASLD risk factor (eg, obesity, dyslipidemia, and hypertension). Each case must have a ground-truth fibrosis stage determined by liver biopsy or expert consensus based on VCTE. To ensure fidelity to real-world clinical presentation, cases will include complete routine clinical data such as age, AST, ALT, platelet count, HbA_1c_, BMI, and creatinine or estimated glomerular filtration rate, along with relevant clinical notes. The simulation case library will be developed using deidentified or synthetic cases derived from representative clinical datasets and prior cohorts, with case selection designed to capture a range of fibrosis risk profiles and clinical presentations. Cases will be curated to ensure clinical realism, balanced representation across fibrosis stages, and inclusion of all variables required for FibroX and comparator assessments.

### Interventions

The intervention arm, known as FibroX-enabled care, will use a simulated EHR-like interface delivered through a secure web-based platform that mirrors the presentation of routine outpatient clinical information. Participants will review standardized simulated cases containing patient demographics, relevant clinical history, laboratory data, and clinical notes. Within this interface, FibroX-enabled care will display key predictive and explanatory information at the point of decision-making. This will include individualized FibroX risk estimates for advanced fibrosis generated using routinely available clinical data. Crucially, the interface presents dual-threshold triage bands aligned with established clinical guidance, classifying patients into 3 actionable categories: rule out, indeterminate (with a pathway to VCTE or other noninvasive testing), and rule in (with a pathway to hepatology referral). In addition, the interface presents case-level SHAP outputs that display the relative contribution of individual clinical variables (eg, age, AST, ALT, platelet count, HbA_1c_, BMI, and kidney function) to the predicted risk of advanced fibrosis. These explanations are presented in a structured visual format, such as ranked feature contributions, alongside the risk estimate and triage category to support clinician interpretation at the point of decision-making. By providing case-level explanations of the key drivers of predicted risk, these SHAP-based outputs are intended to support clinician interpretation of the model’s recommendations, increase confidence in triage decisions, and facilitate more consistent application of guideline-concordant referral pathways compared with usual care, where risk estimation is often implicit and variable.

The comparator arm, designated as usual care, will present the same standardized clinical case information within the simulated EHR-like interface but without FibroX risk estimates, SHAP explanations, or AI-based triage support. Participants in this arm will make diagnostic and management decisions based on routine clinical judgment alone. Optional access to a basic FIB-4 calculator will be provided, reflecting common clinical practice for noninvasive fibrosis risk stratification. FIB-4 is a widely used blood-based fibrosis score derived from age, AST, ALT, and platelet count to estimate the likelihood of advanced fibrosis [23].

### Outcomes

The primary end points of this pilot study are the feasibility and usability of FibroX-enabled care within diabetes-focused clinical workflows. Feasibility will be assessed using recruitment and study completion rates, with prespecified targets of enrolling at least 70% of eligible clinicians and achieving a completion rate of at least 85% among enrolled participants. Completion will be defined as participants completing both simulation periods, including all assigned cases and postperiod surveys. Participants who do not complete both periods or required assessments will be classified as incomplete. Usability will be evaluated using the System Usability Scale (SUS), with a prespecified success threshold of 70 or higher.

Secondary end points include workflow efficiency and preliminary diagnostic performance. Workflow efficiency will be assessed by median time per case, with an anticipated threshold of 3.5 minutes or less. Preliminary diagnostic performance outcomes will include sensitivity, specificity, and the area under the receiver operating characteristic curve for the detection of MASLD-associated advanced fibrosis. Calibration will be examined using intercept, slope, and graphical assessment of predicted versus observed risk.

Exploratory end points will include clinician trust and cognitive workload, assessed using the AI trust scale and National Aeronautics and Space Administration Task Load Index (NASA-TLX), respectively, as well as implementation-related outcomes, including the proportion of guideline-concordant referrals and net reclassification improvement. Qualitative debrief interviews will further explore clinician understanding, interpretability, and perceived usefulness of the SHAP-based explanations, including how these outputs influence decision-making and trust in the model. These measures are intended to provide pilot estimates and inform the design, end point selection, and sample size considerations for a future definitive trial.

At a high level, this pilot will be considered successful if predefined feasibility and usability thresholds are met, including adequate recruitment and completion rates and a SUS score of 70 or higher, alongside signals of acceptable workflow efficiency and improved or comparable diagnostic performance relative to usual care. These criteria are intended to determine whether progression to a larger, real-world trial is justified.

### Participant Timeline and Study Schedule

This health care provider–level crossover pilot is planned over a 12-month period. Months 0 to 2 will be used for finalizing the platform and completing quality assurance of the simulated case library. The first study period will then be conducted during months 2 to 4, followed by a 1-week washout period to reduce potential carryover effects. The second study period will take place during months 4 to 6. After completion of both periods, participants will complete postperiod surveys assessing usability, trust, and cognitive workload, followed by brief semistructured debrief interviews. The remaining months will be dedicated to data cleaning, analysis, qualitative review, and final reporting.

### Sample Size

At least 36 health care providers (32 cases/health care provider) yield 1150 to 1280 paired decisions. Under pilot assumptions (accuracy 0.60 usual care vs 0.75 FibroX), McNemar test provides >80% power to detect a 15-point improvement at α=.05 after accounting for health care provider–level clustering via a conservative design effect. Precision estimates for feasibility and usability metrics are expected to be narrow enough to inform a subsequent multicenter trial.

### Randomization, Allocation Concealment, and Implementation

**Figure 2:**
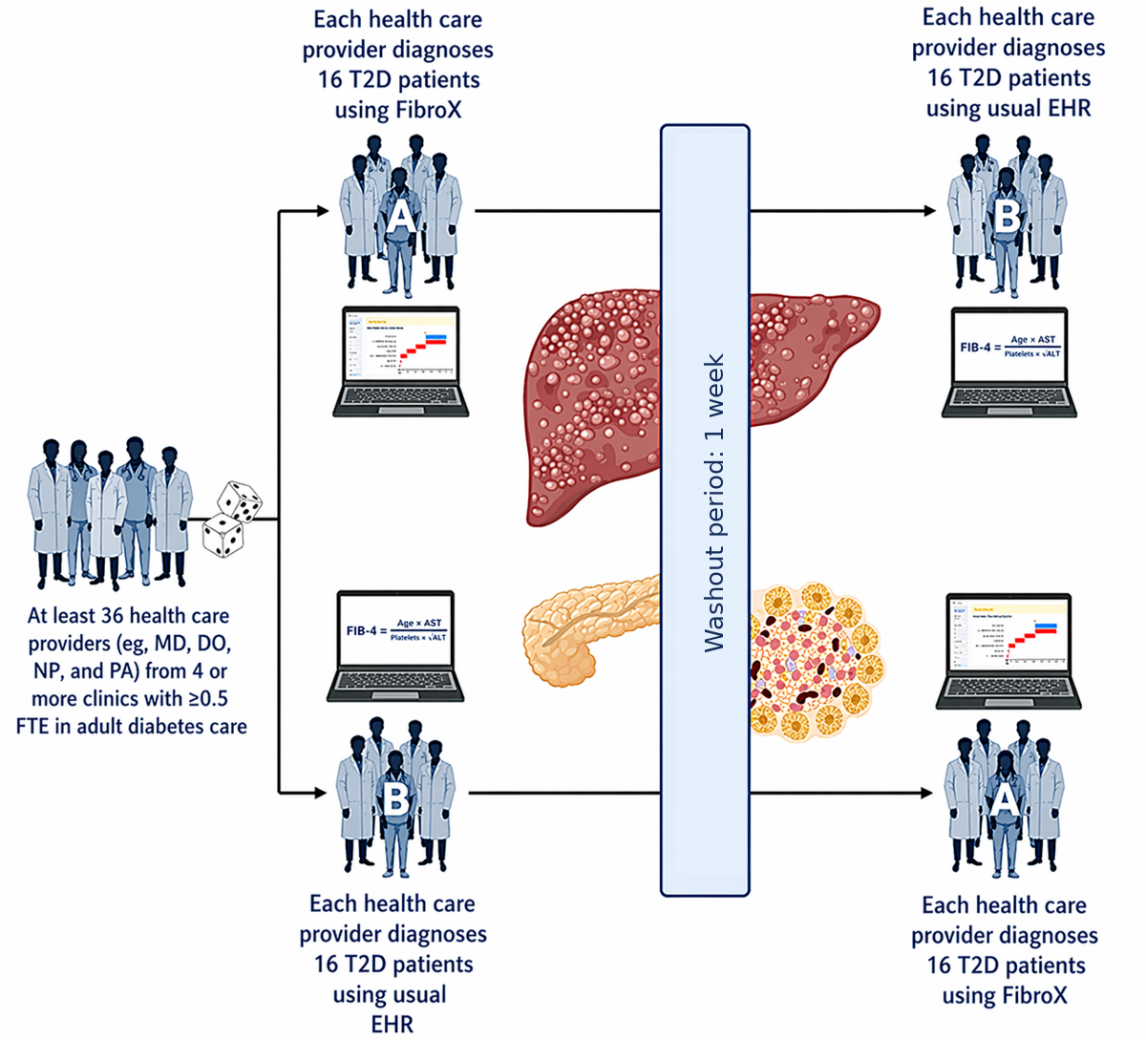
FibroX health care provider–level crossover randomized controlled trial pilot design. DO: doctor of osteopathic medicine; EHR: electronic health record; FTE: full-time equivalent; MD: doctor of medicine; NP: nurse practitioner; PA: physician assistant; T2D: type 2 diabetes.

Health care providers will be randomized 1:1 to AB (FibroX then usual care) or BA (usual care then FibroX) using a computer-generated schedule prepared by a statistician not involved in recruitment (Figure 2). Allocation will be concealed via a secure, centralized randomization module until assignment. The research coordinator enrolls participants and implements sequence assignments.

### Blinding

Blinding of health care providers is not feasible due to visible tool features. Outcome assessors and data analysts are masked to sequence assignment; datasets use anonymized identifiers.

### Data Collection Methods

The platform automatically logs case navigation, decisions, timestamps, and use of FibroX features and overrides. Clinicians will not receive case-level performance feedback during either simulation period. Feedback, if provided, will be deferred until completion of the study period to minimize within-period learning effects. Surveys (SUS, AI trust, and NASA‑TLX) are administered electronically at the end of each period. Qualitative debriefs are audio-recorded and transcribed for thematic analysis. Completeness checks and range validations occur at ingestion.

### Statistical Analysis

All analyses will follow an intention-to-treat principle, including all health care provider decisions regardless of sequence assignment. The primary comparison of diagnostic accuracy between FibroX-enabled care and usual care will use paired analyses to account for the crossover design. Sensitivity and specificity for detecting MASLD-associated advanced fibrosis will be compared using McNemar test for paired proportions. Overall discrimination will be assessed by calculating the area under the receiver operating characteristic curve for each condition, and calibration will be evaluated through intercept, slope, and calibration plots to determine agreement between predicted probabilities and observed outcomes.

To account for clustering at the health care provider level and the crossover design, mixed-effects logistic regression models will be used, specifying health care provider as a random effect and study condition, study period, and sequence assignment as fixed effects. Sensitivity analyses will also assess whether diagnostic performance differs by sequence assignment or period, which may suggest residual carryover or learning effects. These models will also adjust for case-level covariates such as age, BMI, and HbA_1c_ to explore potential confounding and heterogeneity. Secondary analyses will include net reclassification improvement to quantify whether FibroX improves assignment of cases into clinically appropriate triage categories compared with usual care. Bootstrap resampling will be used to generate CIs for net reclassification improvement and calibration metrics, ensuring robust estimation under small-sample conditions.

Feasibility outcomes, including recruitment and completion rates, will be summarized descriptively with proportions and 95% CIs. Usability and trust scores from the SUS and AI trust scale will be analyzed using paired *t* tests or Wilcoxon signed-rank tests, depending on distributional assumptions. Cognitive workload scores from NASA-TLX will be compared similarly. Subgroup analyses will explore whether diagnostic performance varies across diabetes-related characteristics such as HbA_1c_, BMI, and duration of diabetes, using interaction terms in mixed-effects models. Missing data are expected to be minimal; if more than 5% of survey data are missing, multiple imputation under a missing-at-random assumption will be applied, with sensitivity analyses conducted using complete-case data. All statistical tests will be 2-sided with an α level of .05, and analyses will be performed using the R (R Foundation for Statistical Computing) or Python (Python Software Foundation) statistical packages.

To aid interpretation of the crossover design, sequence, period, and potential carryover effects will be explicitly examined. Sequence effects (AB vs BA) will assess whether the order of exposure to FibroX influences outcomes. Period effects will evaluate whether outcomes differ between the first and second study periods, independent of the intervention, which may reflect learning or temporal effects. Potential carryover effects will be explored by assessing interaction terms between intervention and study period and by comparing outcomes across sequences; a significant difference by sequence or interaction with period would suggest residual carryover. If meaningful carryover effects are detected, sensitivity analyses restricted to first-period data will be performed.

### Monitoring, Harms, and Auditing

Given simulated, minimal-risk procedures, no independent monitoring board will be convened. The research coordinator will track enrollment, completion, protocol adherence, and technical issues weekly. Anticipated risks are limited to time burden and performance discomfort; scheduling flexibility, planned breaks, and aggregated reporting mitigate these risks.

### Ethical Considerations

Institutional review board (IRB) approval has been obtained from the Yale University IRB (HIC 2000027433). Electronic informed consent will be obtained from all health care providers prior to participation. In addition, protocol amendments will be documented, versioned, and communicated to the IRB and trial registry before implementation when required. Participants will not receive financial compensation for participation. All data will be handled in accordance with institutional privacy and confidentiality policies, with secure storage on encrypted servers and restricted access to authorized study personnel only.

All data reside on Health Insurance Portability and Accountability Act (HIPAA)–compliant, access‑controlled servers with encryption at rest and in transit, role‑based permissions, and audit trails. A master linkage file is stored separately. Only authorized staff have access to identifiable enrollment information; analysis datasets are deidentified. Data retention follows institutional policy and sponsor requirements.

### Study Status and Timeline

This manuscript describes a prospective study protocol. At the time of submission, participant recruitment and data collection had not yet commenced. The trial was registered with ClinicalTrials.gov (NCT07305324). Additional study status details and anticipated milestones are summarized in the Results section.

### Reporting and Dissemination

This protocol has been developed in accordance with the SPIRIT (Standard Protocol Items: Recommendations for Interventional Trials) 2025 statement and the SPIRIT-AI extension for clinical trial protocols involving AI intervention [24,25]. The study schedule is presented in Table 1, in accordance with the SPIRIT framework. The completed SPIRIT checklist is provided as Multimedia Appendix 1. Upon completion of the study, findings will be submitted for presentation at a relevant professional society meeting and for publication in a peer-reviewed journal. The final results manuscript will adhere to CONSORT (Consolidated Standards of Reporting Trials) guidelines [26,27]. In addition, lay summaries will be prepared for participating sites and disseminated through professional networks to facilitate knowledge translation and support future implementation. Deidentified datasets and analysis code will be made available upon reasonable request following publication and appropriate data‑sharing agreements. Findings will be disseminated via conferences, a peer‑reviewed manuscript, and lay summaries for participating sites.

**Table 1:** Study schedule of enrollment, interventions, and outcome assessments (SPIRIT [Standard Protocol Items: Recommendations for Interventional Trials] framework).

Domain and procedure				Enrollment		Allocation		Intervention period 1		Washout		Intervention period 2		Closeout
				T–2 weeks to T0^a^		T0		T1^b^ (1-2 wk)		T2^c^ (1 wk)		T3^d^ (1-2 wk)		T4^e^
**Enrollment**														
	Eligibility screen and recruitment		✓											
	Informed consent		✓											
	Orientation and technical competency check		✓											
**Allocation**														
	Randomization to sequence (AB^f^ or BA^g^)				✓									
**Intervention**														
	FibroX-enabled care (condition A)						✓^h^				✓^i^			
	Usual care (condition B)						✓^i^				✓^h^			
**Assessments**														
	**Feasibility outcomes**													
		Decision time (workflow efficiency)					✓				✓			
		Usability (SUS^j^ score)					✓				✓			
		Trust in AI^k^ (AI trust scale)					✓				✓			
		Cognitive workload (NASA-TLX^l^)					✓				✓			
	**Effectiveness outcome**													
		Diagnostic accuracy (sensitivity, specificity, and AUROC^m^)					✓				✓			
	**Secondary outcomes**													
		Referral rates, confidence, and override rates					✓				✓			
		Qualitative debrief interview (barriers and facilitators)											✓	
		Final data verification and feedback											✓	

^a^T0: baseline or randomization.

^b^T1: first intervention period.

^c^T2: washout.

^d^T3: second intervention period.

^e^T4: study closeout.

^f^Sequence AB: FibroX-enabled care in period 1 followed by usual care in period 2.

^g^Sequence BA: usual care in period 1 followed by FibroX-enabled care in period 2.

^h^In Sequence AB.

^i^In Sequence BA.

^j^SUS: System Usability Scale.

^k^AI: artificial intelligence.

^l^NASA-TLX: National Aeronautics and Space Administration Task Load Index.

^m^AUROC: area under the receiver operating characteristic curve.

## Results

IRB approval has been obtained. At the time of manuscript submission, participant recruitment had not yet commenced. Preparatory activities, including platform finalization, simulation interface testing, and case library validation, were ongoing in advance of the anticipated study start date of June 15, 2026. Primary completion is anticipated on May 15, 2027, with overall study completion anticipated on June 15, 2027.

## Discussion

### Anticipated Findings

This pilot trial addresses a critical gap in diabetes care: the underdiagnosis of MASLD-associated advanced liver fibrosis despite its strong association with adverse hepatic and cardiovascular outcomes. Current noninvasive tools underperform in T2D populations, and workflow barriers further limit guideline-concordant screening. Because FibroX is not currently used in routine clinical practice, this study evaluates its feasibility, usability, and preliminary diagnostic effectiveness within simulated clinical workflows before progression to real-world implementation. A health care provider–level randomized crossover simulation design was chosen to ensure each clinician serves as their own control, improving internal validity and reducing between–health care provider variability while enabling rigorous paired analyses. Simulation with deidentified or synthetic cases minimizes risk and isolates decision performance from external clinical confounders while preserving the EHR-like context needed to assess workflow integration. This approach allows preliminary evaluation of feasibility, usability, and diagnostic performance in a controlled setting before progression to real-world clinical testing. In addition to improving risk estimation, the inclusion of SHAP-based explanations is designed to make model outputs more interpretable and actionable, potentially supporting more consistent and guideline-aligned decision-making compared with usual care.

### Comparison to Prior Work

Prior studies of machine learning in MASLD and fibrosis risk stratification have largely focused on retrospective model development and diagnostic performance, with comparatively less attention to clinician-facing usability, workflow integration, and implementation [19,21,28]. Explainable approaches such as SHAP have been proposed to improve interpretability, but prospective evaluation of how such tools influence clinical decision-making remains limited [29,30]. In this context, this pilot study extends prior work by moving beyond retrospective validation to assess FibroX as a clinician-facing decision support tool within a simulated workflow environment.

### Strengths and Limitations

This study has several strengths, including its randomized crossover design, within-clinician comparison, standardized simulated cases, and explicit assessment of feasibility, usability, and preliminary diagnostic performance. At the same time, it has limitations inherent to simulation-based designs, including the absence of real-world workflow pressures, competing clinical demands, and direct patient interaction. These features make the study well-suited for an initial pilot evaluation, but clinician behavior in simulated scenarios may not fully reflect decision-making in routine practice. Future research will therefore need to validate these findings in real clinical settings, assess longer-term implementation and outcomes, and examine scalability across diverse health systems, including resource-limited environments where MASLD burden is high.

### Future Work

If FibroX demonstrates acceptable feasibility, usability, and preliminary diagnostic performance in this pilot setting, the next step will be a larger pragmatic study in real-world clinical practice to evaluate workflow impact, referral patterns, and diagnostic effectiveness under routine care conditions. Such work should also assess generalizability across diverse practice settings, including community clinics and resource-limited environments, examine longer-term implementation outcomes, and further evaluate whether SHAP-based explanations improve interpretability and clinician trust in routine care.

### Conclusions

In conclusion, this pilot will provide early evidence on the feasibility, usability, and preliminary clinical utility of FibroX within simulated diabetes care workflows. The findings will help determine whether further real-world evaluation of this explainable AI approach is warranted for MASLD risk stratification in people with T2D.

## Appendix

Multimedia Appendix 1 SPIRIT checklist.

## Abbreviations

AI: artificial intelligence
ALT: alanine aminotransferase
AST: aspartate aminotransferase
CONSORT: Consolidated Standards of Reporting Trials
EHR: electronic health record
FIB-4: Fibrosis-4 Index
HbA_1c_: hemoglobin A_1c_
HIPAA: Health Insurance Portability and Accountability Act
IRB: institutional review board
MASLD: metabolic dysfunction–associated steatotic liver disease
NASA-TLX: National Aeronautics and Space Administration Task Load Index
SHAP: Shapley Additive Explanations
SPIRIT: Standard Protocol Items: Recommendations for Interventional Trials
SUS: System Usability Scale
T2D: type 2 diabetes
VCTE: Vibration-Controlled Transient Elastography

## Data Availability

As this study is a protocol involving simulated clinical cases, no real patient-level data will be generated. Deidentified study data, including aggregated decision outcomes and analysis code, will be made available from the corresponding author upon reasonable request following study completion and publication, subject to institutional policies and data use agreements. Any materials necessary to reproduce the simulation environment or analysis framework may also be shared upon request where feasible.
